# Independent impacts of aging on mitochondrial DNA quantity and quality in humans

**DOI:** 10.1186/s12864-017-4287-0

**Published:** 2017-11-21

**Authors:** Ruoyu Zhang, Yiqin Wang, Kaixiong Ye, Martin Picard, Zhenglong Gu

**Affiliations:** 1 Division of Nutritional Sciences, Cornell University, Ithaca, NY 14853 USA; 2 Department of Biological Statistics and Computational Biology, Cornell University, Ithaca, NY 14853 USA; 3 Department of Psychiatry, Division of Behavioral Medicine, Department of Neurology and Columbia Translational Neuroscience Initiative, Columbia Aging Center, Columbia University Medical Center, New York, NY 10032 USA

**Keywords:** Aging, Heteroplasmy, mtDNA copy number, Whole genome sequencing

## Abstract

**Background:**

The accumulation of mitochondrial DNA (mtDNA) mutations, and the reduction of mtDNA copy number, both disrupt mitochondrial energetics, and may contribute to aging and age-associated phenotypes. However, there are few genetic and epidemiological studies on the spectra of blood mtDNA heteroplasmies, and the distribution of mtDNA copy numbers in different age groups and their impact on age-related phenotypes. In this work, we used whole-genome sequencing data of isolated peripheral blood mononuclear cells (PBMCs) from the UK10K project to investigate in parallel mtDNA heteroplasmy and copy number in 1511 women, between 17 and 85 years old, recruited in the TwinsUK cohorts.

**Results:**

We report a high prevalence of pathogenic mtDNA heteroplasmies in this population. We also find an increase in mtDNA heteroplasmies with age (*β* = 0.011, *P* = 5.77e-6), and showed that, on average, individuals aged 70-years or older had 58.5% more mtDNA heteroplasmies than those under 40-years old. Conversely, mtDNA copy number decreased by an average of 0.4 copies per year (*β* = −0.395, *P* = 0.0097). Multiple regression analyses also showed that age had independent effects on mtDNA copy number decrease and heteroplasmy accumulation. Finally, mtDNA copy number was positively associated with serum bicarbonate level (*P* = 4.46e-5), and inversely correlated with white blood cell count (*P* = 0.0006). Moreover, the aggregated heteroplasmy load was associated with blood apolipoprotein B level (*P* = 1.33e-5), linking the accumulation of mtDNA mutations to age-related physiological markers.

**Conclusions:**

Our population-based study indicates that both mtDNA quality and quantity are influenced by age. An open question for the future is whether interventions that would contribute to maintain optimal mtDNA copy number and prevent the expansion of heteroplasmy could promote healthy aging.

**Electronic supplementary material:**

The online version of this article (10.1186/s12864-017-4287-0) contains supplementary material, which is available to authorized users.

## Background

Mitochondria play a central role in cellular energy metabolism, as well as in a range of other cellular activities, such as calcium signaling, iron homeostasis, hormone synthesis, and programmed cell death [1–3]. Mitochondria differ from all other organelles in animals in having their own DNA (mitochondrial DNA, mtDNA), which in humans encodes 37 genes: 22 tRNAs, 2 rRNAs and 13 protein subunits of the electron transport chain and Complex V/ATP synthase. Although they contribute only ~1% of the mitochondrial proteome, the 13 mtDNA-encoded proteins are nevertheless essential for mitochondrial oxidative phosphorylation and cellular energetics [4]. A single mammalian cell hosts hundreds to thousands of copies of mtDNA, which are thought to have played a critical role in the evolution of mammalian genomic complexity [5]. Because of its multi-copy nature, spontaneous mtDNA mutations often affect only a small proportion of the cell’s mtDNA, a state termed *heteroplasmy*. In contrast, if all mtDNA molecules harbor a specific mutation, it is said to be in a state of *homoplasmy*. mtDNA heteroplasmy is implicated in several human diseases, in which the ratio of mutated to wild-type mtDNA is critical in determining whether a specific mutation is deleterious [2, 6, 7]. In previous studies, we demonstrated that even in healthy adults, low-frequency heteroplasmies with high pathogenic potential were common [8].

In addition to mtDNA mutation burden, the number of mtDNA molecules per cell, or “mtDNA copy number”, is also strictly regulated, ensuring that mitochondria can generate appropriate levels of energy and intracellular signals to maintain normal cellular functions. Altered mtDNA copy number has been shown to be involved in age-related diseases, including cancer, neurodegeneration disorders, and diabetes [9–11]. In the general population, mtDNA copy number measured in peripheral blood has also been shown to be associated with a variety of physiological phenotypes, and to be linked with aging and mortality [12, 13]. For example, higher mtDNA copy number was linked with better physical and mental health status in aged populations [12]. It has been speculated that both mtDNA heteroplasmy and copy number may contribute to the aging process, but the effects of mtDNA heteroplasmy and copy number were only discussed separately, thus remaining inconclusive in humans [14].

Aging is commonly characterized as a time-dependent progressive loss of physiological integrity, leading to impaired function and increased vulnerability to death [14]. One important factor in aging is the accumulation of DNA damage over time [15]. mtDNA has been considered a major target of aging-associated mutation accumulation, possibly because it experiences higher oxidative damages, more turnover, and has lower replication fidelity compared to nuclear DNA (nDNA) [16–18]. Mice carrying elevated mtDNA mutation burden present premature signs of aging including hair loss, kyphosis, and premature death (lifespan shortened by up to 50%) [19, 20]. In human studies, mtDNA heteroplasmy incidence increases with age [21–23], while lower mtDNA copy number has been reported in aged populations [12, 24]. Ding et al. reported an trend of increased heteroplasmies and decreased mtDNA copy number with age in their study population [25]. However, previous studies were limited in one or more ways: i) limited power in detecting low-to-medium frequency heteroplasmies in blood due to low sequencing depth; ii) relatively small sample sizes, limiting statistical power; iii) small age range; iv) whole blood as the source of DNA, which contains several sources of contaminants for mtDNA analysis; and/or v) assessing either mtDNA mutation or copy number, but not both in the same biological samples. Thus, it is largely unknown whether the impacts of age on mtDNA mutation burden and on copy number are independent from each other.

Whole genome sequencing (WGS) data allows us to study mtDNA heteroplasmy and copy number simultaneously. Previous large-scale studies of mtDNA heteroplasmy or copy number mostly used sequencing data of total genomic DNA extracted from transformed cell lines or whole blood. It is possible that during cell line transformation, both mtDNA heteroplasmy and copy number could undergo marked changes [26]. Moreover, estimating mtDNA copy number from WGS data relies on the ratio of sequencing reads for the mitochondrial and nuclear genomes extracted from the biological samples. There are numerous factors in the whole blood that can bias the estimation of mtDNA copy number. For example, platelets have high mtDNA content, but lack nuclear DNA; mtDNA from platelets therefore artificially raises the estimated mtDNA copy number from the whole blood [27, 28]. In the current study, we focused our analysis on WGS data of isolated platelet-free peripheral blood mononuclear cells (PBMCs) DNA obtained from the UK10K project TwinsUK cohort, which includes individuals ranging from 17 to 85 years of age [29]. The TwinsUK is a cohort with WGS data for more than 1500 generally healthy female individuals with phenotypic data. The resulting mtDNA-phenotypic dataset is one of the largest available in a general human population for analysis on the relationship between age and mtDNA heteroplasmy and copy number. Our analyses reveal that these two mtDNA properties are significantly correlated with age, and that these age effects were independent. Our results further indicate that mtDNA copy number and heteroplasmy load were significantly associated with age-related physiological parameters in this population, suggesting potential pathways by which age-related mtDNA alterations may impact the aging process.

## Methods

### Data access permission

Data used in this study was obtained from UK10K project, “UK10K Data Access Agreement” was approved by UK10K Data Access Officer.

### mtDNA variation identification and haplogroup assignment

Whole genome sequencing and subsequent read mapping of the TwinsUK cohorts were accomplished by the UK10K project [29]. Briefly, DNA (1–3 μg) extracted from PBMCs was sheared to 100–1000 bp, and sheared DNA was sent to Illumina paired-end DNA library preparation. After size selection (300–500 bp insert size), the DNA library was sequenced by the Illumina HiSeq platform with paired-end read lengths of 100 bp. Sequencing reads mapping to the mitochondrial genome were extracted from indexed bam files to identify heteroplasmy in each individual. Retrieved reads were re-mapped to the combined human genome, hg19 for the nuclear genome and the revised Cambridge Reference Sequence (rCRS) for the mitochondrial genome, using bowtie2 [30]. Read pairs with proper orientation and less than 5 mismatches were retained from the mapping results. The nuclear genome contains some regions with high similarity to part of the mtDNA (nuclear mitochondrial DNA, abbreviated as NUMTs). To minimize the effect of NUMTs for heteroplasmy calling, we further required the retained reads to be uniquely mapped to the mitochondrial genome. Filtered reads were further processed following the GATK best practice workflow, including Mark duplicates (duplicated reads were removed), Indel realignment, and Base quality score recalibration steps. Homoplasmies were identified using GATK HaplotypeCaller and GenotypeGVCFs [31]. Haplogroups were assigned using homoplasmic variants identified from each sample by HaploGrep2 [32]. To identify heteroplasmy, sequencing data for each position of the mitochondrial genome was extracted by Samtools mpileup [33], and bases were further filtered by sequencing quality (> = 20). Heteroplasmy was then identified with the following criteria: 1) Sequencing coverage >200. 2) Minor allele frequency > = 2%. 3) Minor allele must be observed at least twice from each strand.

### Potential cross-sample contamination inspection

Potential cross-sample contamination was assessed in the UK10K project’s original data processing by VerifyBamID [34] and “fraction skewed hets” [29]. Potential contaminated samples were already removed from the dataset. However, to be more conservative, we further tested contamination using mtDNA sequencing data, which has better sensitivity than nuclear DNA variants based methods. We evaluated potential contamination by 2 criteria 1) if a sample had extremely high heteroplasmy number (Q3 + 1.5IQR rule); by this criterion, samples having more than 8 heteroplasmies were suspected to be contaminated. 2) We constructed two consensus mtDNA sequences for each sample, one covering the major alleles at heteroplasmic sites, the other covering minor alleles. A sample was suspected to be contaminated if these two consensus sequences belonged to different haplogroups. If a sample met both criteria, we would recognize it as contamination and remove it from further analysis.

### Annotation of mtDNA variants

Heteroplasmy and homoplasmy were annotated by customized scripts. Pathogenic potential of variants was predicted using Combined Annotation-Dependent Depletion (CADD) score (version 1.3) [35]. The CADD score integrated many diverse annotations of variants, including functionality, pathogenicity, experimentally measured effects etc., into a single score, which has been shown to have better performance than other predictive methods such as Grantham, SIFT and PolyPhen. As recommended, a scaled CADD score of 15 was used to define the pathogenic mutations. To avoid the bias of an arbitrary cutoff, a series of cutoffs from 12 to 22 were also applied to evaluate the variants’ pathogenicity. The disease associated mtDNA mutations were obtained from the MITOMAP database [36].

### mtDNA copy number estimation

Whole genome sequencing data of the study population were retrieved from the UK10K project [29]. To estimate mtDNA copy number, we further filtered mapped reads by the following criteria: 1) Mapping quality >20. 2) Reads were not PCR duplicates. 3) Mismatches <5. We proceeded with qualified reads for subsequent calculation. Sequencing coverage of each site in the reference genome was calculated by the Samtools mpileup function [33]. The average sequencing coverage was then calculated for each autosomal DNA and mtDNA locus. mtDNA copy number of each individual was further estimated based on Eq. 1 and Eq. 2. It has been shown that NUMTs have a negligible impact on mtDNA copy number estimation by this method [37].

1 $$ \frac{\mathit{\mathsf{mtDNAaveragecoverage}}}{\mathit{\mathsf{autosomal}}\;\mathit{\mathsf{DNA}}\;\mathit{\mathsf{averagecoverage}}}=\frac{\mathit{\mathsf{mtDNAcopies}}}{\mathit{\mathsf{autosomal}}\;\mathit{\mathsf{DNA}}\;\mathit{\mathsf{copies}}} $$

2 $$ \mathit{\mathsf{mtDNAcopynumber}}=\frac{{\mathsf{2}}^{\ast}\mathit{\mathsf{mtDNAcoverage}}}{\frac{\mathsf{1}}{\mathsf{2}\mathsf{2}}\sum \limits_{\mathit{\mathsf{i}}=\mathsf{1}}^{\mathsf{2}\mathsf{2}}\mathit{\mathsf{autosomalcoverage}}} $$

### Association testing for mtDNA copy number and heteroplasmy

Linear regression was carried out to test the association of mtDNA heteroplasmy with age, as well as copy number with age separately. To conveniently test the independence of the effects of age on mtDNA heteroplasmy and copy number, we performed a linear regression using age as response variable, and mtDNA copy number and heteroplasmy number as independent variables in the basic model (Eq. 3). WBC count and platelet count were reported to affect mtDNA copy number [24], thus these two factors were also included as covariates in the regression model for further analysis (Eq. 4). Down sampling was carried out by randomly sampling 0.06 million mtDNA reads from each individual and identifying heteroplasmy following the same criteria. The association of mtDNA copy number with mtDNA heteroplasmy number was assessed by linear regression, with age and mean nuclear coverage included as covariates. The influence of mtDNA variants (heteroplasmy, homoplasmy, haplogroup) on mtDNA copy number was tested by linear regression, with age and mean nuclear coverage included as covariates. For homoplasmy, variants present in more than 1% individuals were tested. The significance level was adjusted for multiple testing. Homoplasmic variants with *P* value <2.69e-4 were considered to be significant.

3 $$ \mathit{\mathsf{Age}}\sim \mathit{\mathsf{mtDNA}}\;\mathit{\mathsf{h}}\mathit{\mathsf{eteroplasmynumber}}+\mathit{\mathsf{mtDNA}\mathsf{copynumber}} $$

4 $$ \mathit{\mathsf{Age}}\sim \mathit{\mathsf{mtDNA}}\;\mathit{\mathsf{h}}\mathit{\mathsf{eteroplasmynumber}}+\mathit{\mathsf{mtDNA}\mathsf{copynumber}}+\mathit{\mathsf{WBC}}\;\mathit{\mathsf{count}}+\mathit{\mathsf{Plateletcount}} $$

### Phenotypic associations of mtDNA copy number and heteroplasmy

We assessed the associations of 32 phenotypes directly measured from blood samples with mtDNA copy number and heteroplasmy number. The details of phenotypic data measurements can be found from the UK10K project [29]. Linear regression models were applied to test for associations, and age was included as a covariate to adjust for its effects on these phenotypes. Significance levels were adjusted by Bonferroni correction. Effects were considered as significant if *P* value <0.0016.

### Mitochondrial heteroplasmy load and SKAT test

The Sequence Kernel Association Test (SKAT) has been shown to have high statistical power under a variety of conditions [38]. We used it to test the association of mtDNA heteroplasmic mutation load with different phenotypes. To do the association test, we first constructed a genotype matrix containing mtDNA heteroplasmy information. Assuming that we have mtDNA sequences for n individuals, and there are, in total, m unique heteroplasmic variants among those individuals, then the genotype matrix could be constructed an n x m matrix X, where the entry a_ij_ in X represents the minor allele frequency of individual i at heteroplasmic site j. To calculate a_ij_, the number of all possible bases (A, T, G, C) with sequencing quality >20 were counted for individual i at site j. If the minor allele count exceeded 5, minor allele frequency would be calculated by dividing the minor allele count by total coverage at the given site, otherwise a_ij_ would be set to 0. We also constructed another genotype matrix X’, whose entries were either 0 or 1. This matrix will only consider whether a given site is heteroplasmic (as 1) or not (as 0), regardless of the minor allele frequency. Notably, the genotype matrices only contained the non-polymorphic heteroplasmies. After constructing the genotype matrix, a linear regression model can be considered (Eq. 5):

5 $$ \mathsf{Y}={\alpha}_{\mathsf{0}}+ C\alpha + X\beta $$

Where C is the covariates matrix, in which we included age, mtDNA copy number, and the top 2 PCs from principle component analysis of population structure. Phenotypes were log transformed to achieve normal distributed residuals. The CADD score of each heteroplasmic variant was used as the weights. This weighting scheme could upweight heteroplasmic variates which are predicted to be more deleterious. The tests were performed using the R package SKAT [38].

## Results

### Mitochondrial heteroplasmy is prevalent in UK10K TwinsUK cohort

The UK10K-cohorts arm [29] provided WGS data for healthy individuals from two British cohorts of European ancestry, namely the Avon Longitudinal Study of Parents and Children (ALSPAC) [39] and TwinsUK [40]. However, the genomic DNA used in these two cohorts was different. In ALSPAC, DNA was extracted from lymphoblastoid cell lines established in vitro, while DNA in TwinsUK was extracted from isolated PBMCs. We compared the mtDNA copy number distribution between the two cohorts and observed a dramatically higher mtDNA copy number in cell line DNA (Additional file 1: Figure S1). The higher mtDNA copy number in the cell line compared to PBMCs is likely attributable to the difference in biological material, rather than a genuine cohort difference. Therefore, in the current study, we only focused on individuals from the TwinsUK cohort for which PBMC DNA was available. In the UK10K project’s original study design, in order to increase the genetic diversity and decrease the sequencing costs, only one individual from each of the twin pairs recruited was sequenced at the whole-genome level. After excluding 73 individuals with potential cross-sample contamination, we retained 1511 individuals for further analysis. The average age of these studied individuals was 55.5 years (SD = 12.8 years), ranging from 17.3 years to 84.5 years.

**Fig. 1:**
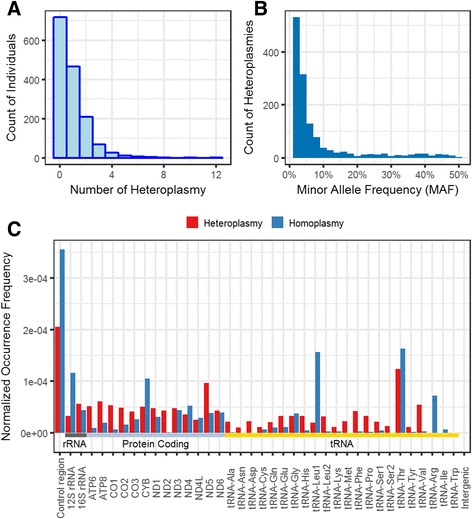
Distribution of heteroplasmy in UK10K TwinsUK cohort. **a** Counts of individuals harboring a specific number of heteroplasmies (0–12 heteroplasmies at MAF > 2% cutoff). More than half of individuals (52.5%) carried at least one heteroplasmy in their genome. **b** Histogram for MAF of all heteroplasmies. 62.7% of heteroplasmies had MAF < 5% and 20.1% had MAF > 10%. **c** Normalized occurrence frequency distribution of heteroplasmies and homoplasmies. The frequency was normalized by the length of the mitochondrial loci. Dark gray, light blue and yellow bars indicate the genes in three different functional categories: rRNA, Protein coding and tRNA, respectively. The distribution of variants was relatively homogeneous among coding regions, except for some regions, such as higher frequency in ND5 (heteroplasmy) and tRNA Thr (heteroplasmy and homoplasmy)

The average sequencing coverage of the mitochondrial genome in these individuals was ~568X, allowing us to reliably identify mtDNA heteroplasmy at 2% minor allele frequency (MAF) cutoff. After applying a series of criteria to filter out low-quality heteroplasmies, we identified 1348 mtDNA heteroplasmies in 1511 individuals; the detailed heteroplasmy information is summarized in Additional file 2: Table S1. 794 (52.5%) individuals harbored at least one heteroplasmy in their mitochondrial genome (Fig. 1a). Most heteroplasmies presented at low-to-medium frequency (62.7% of heteroplasmies have MAF < 5%, Fig. 1b). The gene-length normalized distribution of heteroplasmy frequency among mtDNA loci is shown in Fig. 1c. The distribution of homoplasmy frequency was also plotted. Heteroplasmies were observed over the entire mitochondrial genome. One exception was the control region, also known as the “hypervariable region” [41], which harbored the highest occurrence (normalized by region length) of both homoplasmic and heteroplasmic variants. Other regions were relatively homogenous with a few exceptions (Additional file 1: Table S2): tRNA-Thr, which is positioned immediately upstream from the control region, had significantly higher frequencies than other tRNA genes in both heteroplasmy and homoplasmy (*P* = 0.00015 and 0.00297, respectively, Chi-squared outlier test). Interestingly, we observed that ND5 had a moderate occurrence frequency in homoplasmy, but significant higher frequency in heteroplasmy than other protein coding genes (*P* = 0.00494, Chi-squared outlier test).

### Mitochondrial heteroplasmy has high pathogenic potential

**Fig. 2:**
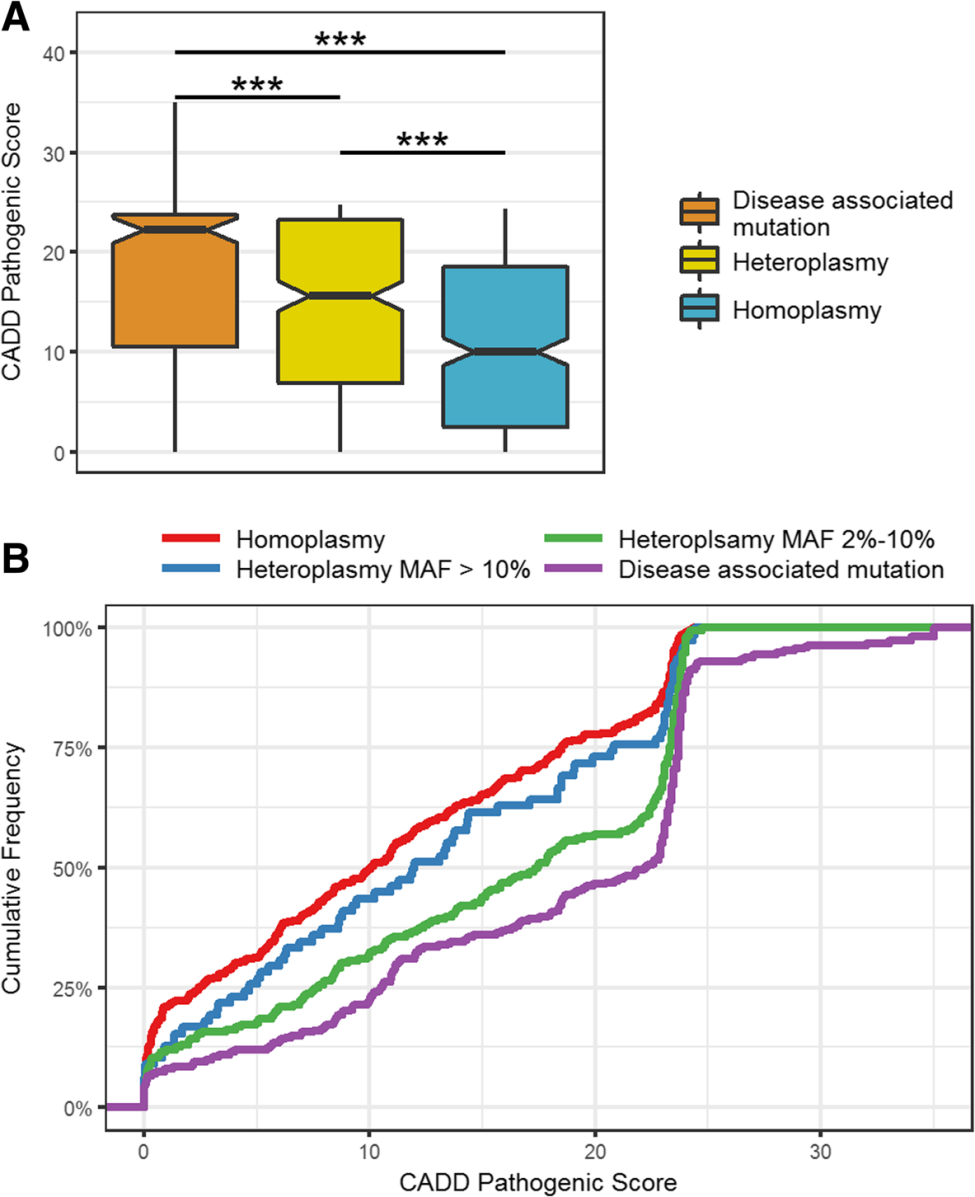
Pathogenic potential for nonsynonymous heteroplasmies. **a** The box plot of CADD pathogenic score for disease associated mutations, nonsynonymous heteroplasmies and nonsynonymous homoplasmies (heteroplasmy and homoplasmy occurring in multiple individuals were counted only once). Heteroplasmies had significant higher pathogenic scores than homoplasmies (*P* = 3.967e-7) although still lower than disease associated mutations (*P* < 2.2e-16). **b** The cumulative distribution of CADD pathogenic scores of disease associated mutation, homoplasmy, low frequency heteroplasmy (MAF 2%–10%) and high frequency heteroplasmy (MAF > 10%). The distribution of low frequency heteroplasmy was close to disease associated mutations, indicating higher pathogenic potential

Among the 1348 heteroplasmies, 192 (14.2%) were previously reported to be associated with diseases. 11.4% of individuals harboring at least one of these diseases-associated heteroplasmies. To further investigate the pathogenicity of the heteroplasmies, combined annotation dependent depletion (CADD) scores [35] were used to predict the potential pathogenicity of nonsynonymous mutations in heteroplasmy and homoplasmy. We also annotated CADD scores for disease-associated mutations (retrieved from MITOMAP [36]) as a comparison. Disease-causing nonsynonymous mutations had a mean pathogenicity score of 17.43; in comparison, the mean CADD score of the 294 unique nonsynonymous heteroplasmic mutations was 14.32, which was significant higher than the 359 unique nonsynonymous homoplasmic mutations (10.84. *P* = 3.967e-7, Welch two sample t-test, Fig. 2a, Additional file 1: Figure S2). As suggested by the CADD score database, CADD score > 15 can be used as a cutoff to define mutations with high possibility to be pathogenic [35]. With this cutoff, the proportion of high pathogenic potential mutations in heteroplasmy was significantly higher than that in homoplasmy (*P* = 0.00825, Chi-square test). With the same criterion, 51.4% of heteroplasmies were high pathogenic potential while only 34.8% of homoplasmic mutations were high pathogenic potential. To avoid the potential bias of the arbitrary cutoff, we also applied a series of CADD score cutoffs from 12 to 22, and heteroplasmy was 1.42 to 1.94 times more likely to be high pathogenic potential than homoplasmy under different cutoffs (Additional file 1: Figure S3), consistent with the notion that more pathogenic mutations are more likely to be eliminated through purifying selection than less deleterious ones [8].

To further investigate this hypothesis, we separated nonsynonymous heteroplasmy into low frequency and high frequency groups using 10% MAF as a cutoff. The low frequency heteroplasmy group had significantly higher CADD scores than the high frequency group (*P* = 0.019, Welch two sample t test). Again, to avoid the potential bias of arbitrary cutoffs, we applied several MAF frequency cutoffs to separate low and high frequency groups (from 5% to 9%), and the difference remained significant until the cutoff was as low as 6%. To visualize this difference, we plotted the cumulative distribution of CADD scores for each group. The distribution of CADD scores for low frequency heteroplasmies approached that of disease-associated mutations, whereas the distribution of high frequency heteroplasmies moved towards that of homoplasmic mutations (Fig. 2b, Additional file 1: Figure S4).

### Mitochondrial heteroplasmy burden increases with age

The mtDNA haplogroup of each individual was identified using Haplogrep2 [32]. In this population, 48.5% (733) of the individuals belonged to H haplogroup, and there were 4 other haplogroups having more than 100 individuals: U (220), K (143), J (135) and T (125). This distribution is typical for a population of predominantly European descent. The haplogroups did not significantly contribute to the heteroplasmy variance (*P* > 0.05 for each haplogroup, Additional file 1: Figure S5), and thus were not considered in subsequent analysis.

mtDNA mutations have been thought to play an important role in aging. To investigate changes of heteroplasmies with age, we first applied linear regression and found that heteroplasmy number increased with age (*β* = 0.011, *P* = 5.77e-6, linear regression. Additional file 1: Figure S6). To better describe the changing heteroplasmy trend during aging, we further divided the 1511 individuals into five age groups. We observed a gradual and consistent increase of heteroplasmy number from the youngest group aged under 40-years to the oldest group aged over 70-years (Table 1). On average, individuals over 70-years old had 1.11 heteroplasmies, significantly higher than individuals under 40-years old (0.70 heteroplasmy, *P* = 0.001593, Welch two sample t test). We also separated heteroplasmy into low-to-medium MAF (2%–5%) and medium-to-high MAF (>5%) intervals, and found that this increasing trend was consistent for heteroplasmy in different MAF intervals. Individuals under 40-years old had 0.41 heteroplasmy with MAF 2%–5% and 0.29 heteroplasmy with MAF >5%, while individuals over 70-years old had 0.68 and 0.43, respectively.

**Table 1:** Heteroplasmy number in different age groups

Age Group	< 40	40–50	50–60	60–70	> 70
Age Mean	30.12	45.24	55.27	64.28	74.07
Age SD	6.41	2.72	2.92	2.94	3.35
Individual count	166	267	464	447	167
Heteroplasmy count^a^	0.70; (44.6%)	0.72; (47.2%)	0.89; (55.0%)	0.98; (53.5%)	1.11; (60.0%)
Heteroplasmy count (MAF 2–5%)	0.41; (28.3%)	0.48; (32.2%)	0.56; (36.9%)	0.62; (38.7%)	0.68; (39.5%)
Heteroplasmy count (MAF > 5%)	0.29; (22.9%)	0.24; (20.6%)	0.33; (28.4%)	0.37;(27.7%)	0.43; (32.3%)

^a^numbers in parentheses indicate the proportion of individuals harboring heteroplasmy with specified MAF cutoffs. An individual can have heteroplasmies in both MAF groups

Next, we evaluated the spectra of heteroplasmy in the five age groups. In all groups, heteroplasmy was predominantly present in protein coding regions, which was not surprising since protein coding sequences account for >67% of mtDNA. However, there was a tendency for the proportion of nonsynonymous heteroplasmies to increase with age. 25.9% of heteroplasmies were nonsynonymous in the under 40-years group, which increased to 28.6% in the over 70-years group, while the proportion of synonymous heteroplasmies did not significantly change (Table 2). Since nonsynonymous mutations are more likely to cause functional consequences than synonymous ones, this increased nonsynonymous proportion, together with the increased absolute heteroplasmy number in older individuals, could suggest that mtDNA integrity, or “quality” deteriorates with age.

**Table 2:** Regional distribution of heteroplasmy in different age groups

	Control region	Intergenic region	rRNA	tRNA	Nonsynonymous	Synonymous
<40	18.1%	0.9%	12.9%	5.2%	25.9%	37.1%
40–50	24.9%	0.5%	13.0%	6.2%	25.9%	29.5%
50–60	27.3%	0.5%	9.9%	3.4%	26.8%	32.1%
60–70	22.0%	0.2%	12.0%	2.5%	28.2%	35.0%
>70	16.8%	0.0%	13.5%	4.9%	28.6%	36.2%

### Age has independent effects on mtDNA heteroplasmy and copy number

**Fig. 3:**
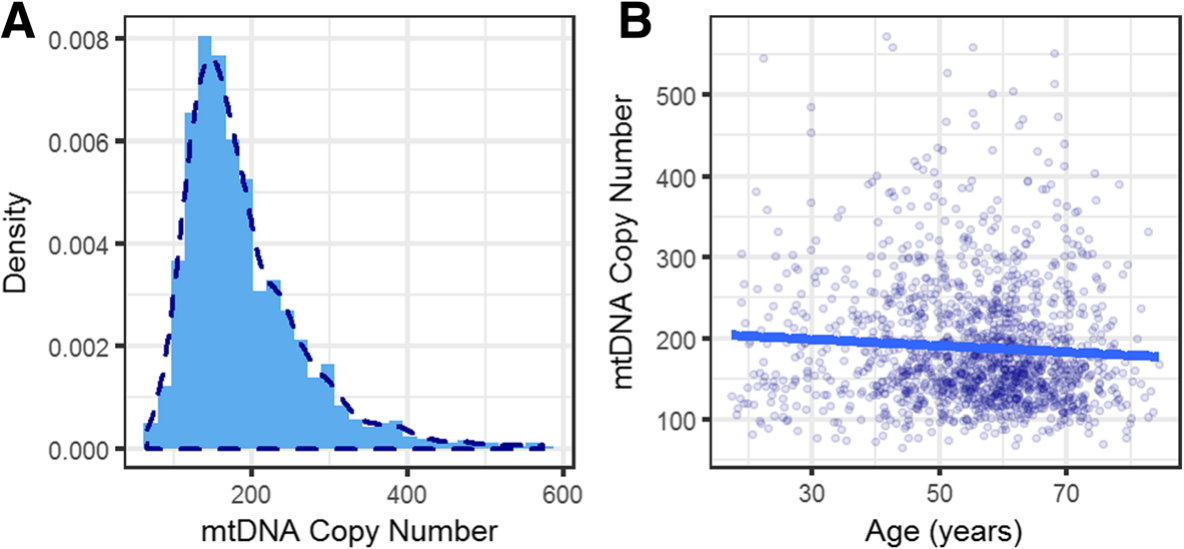
Distribution of mtDNA copy number in the UK10K Twins cohort and its association with age. **a** mtDNA copy number was estimated using WGS data by comparing the mean sequencing coverage of mtDNA and nDNA. The distribution of mtDNA copy number was positively skewed, and most individuals had moderate numbers of mtDNA (mean 169 and median 188). **b** mtDNA copy number was negatively correlated with age (*β* = −0.395, *P* = 0.00972). Blue line represents the linear regression line. For every 10 years, mtDNA copy number decreases about 4 copies

Because mtDNA heteroplasmy level can be affected by copies of mtDNA in blood, the age-related increase of heteroplasmy may reflect the consequences of decreased mtDNA copy number in older individuals [37]. We therefore investigated whether age acted on mtDNA heteroplasmy and copy number independently by incorporating data on heteroplasmy and copy number, simultaneously, in our analytical models with age. In normal human cells, there are two fixed copies of the nuclear genome, and therefore the ratio of average WGS sequencing coverage for mitochondrial and nuclear genomes can be used to estimate mtDNA copy number. Assuming that autosomal and mtDNA are processed and sequenced with no significant difference, average sequencing coverage should be proportional to DNA copy number for autosomal and mtDNA (Eq. 1), thus mtDNA copy number can be estimated using Eq. 2. By this method, we observed a broad range of mtDNA copy number among these individuals (Fig. 3a), from 65 to 573, with mean 169 and median 188. The distribution of mtDNA copy number was positively skewed (*P* < 2.2e-16, D’Agostino’s test), with a coefficient of skewness of 1.55. Our results showed that mtDNA copy number and age were negatively correlated (*β* = −0.395, *P* = 0.00972, linear regression, Fig. 3b). For every 10 years, mtDNA copy number decreases about 4 copies. Similar to mtDNA heteroplasmy, mtDNA copy number was also not significantly affected by haplogroups (Additional file 1: Figure S7).

We next asked whether the effects of age on mtDNA copy number and mtDNA heteroplasmy were independent from one another. We tested this hypothesis by performing a linear regression between age and mtDNA copy number/heteroplasmy number (Eq. 3). In this regression model, both copy number and heteroplasmy number showed significant associations with age (Table 3), suggesting that age-related mtDNA copy number decrease and heteroplasmy increase are independent.

**Table 3:** Correlation of age with mtDNA heteroplasmy number and copy number

Parameter	Parameter Estimate	SE	*P* Value
mtDNA heteroplasmy number	1.185	0.271	1.27e-5 ***
mtDNA copy number	−0.010	0.004	0.0228 *

Significance level (* *P* < 0.05, ** *P* < 0.01 and *** *P* < 0.001)

In addition, since DNA was extracted from blood cells, and WBC count and platelet count were reported to correlate with age and mtDNA copy number [24], we further included WBC count and platelet count as covariates (Eq. 4). These additional adjustments for possible confounding factors did not qualitatively alter the associations obtained in the basic model (Table 4). Additionally, because it was more likely to identify heteroplasmies in individuals with high mtDNA sequencing coverage, as a sanity check, we down sampled mtDNA sequencing reads in each individual to 0.06 million mtDNA reads (corresponding to mtDNA sequencing coverage of ~ 360X) and identified heteroplasmies at 2% MAF cutoff. The independent effects of age on mtDNA heteroplasmy and copy number were still significant in the down-sampled data (Additional file 1: Table S3, S4), confirming the robustness of this finding across the whole population.

**Table 4:** Correlation of age with mtDNA heteroplasmy number and copy number, adjusting for WBC and platelet counts

Parameter	Parameter Estimate	SE	*P* Value
mtDNA heteroplasmy number	0.901	0.252	0.00037 ***
mtDNA copy number	−0.013	0.004	0.00122 **
White blood cell count	−0.301	0.176	0.08787
Platelet count	0.009	0.005	0.08590

Significance level (* *P* < 0.05, ** *P* < 0.01 and *** *P* < 0.001)

### Mitochondrial DNA copy number is associated with number of heteroplasmies

**Fig. 4:**
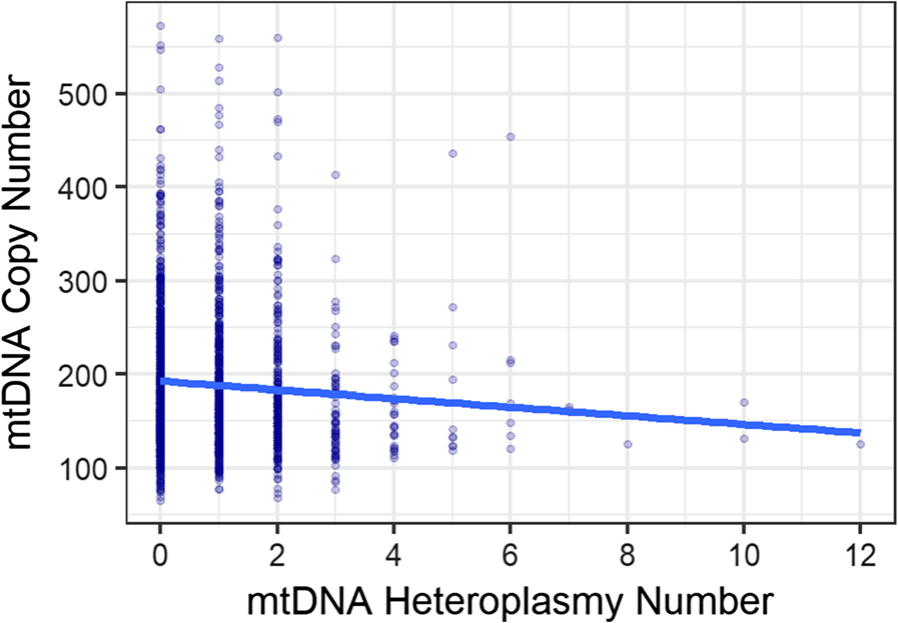
Association between mtDNA heteroplasmy number and copy number. mtDNA copy number was significantly associated with the total heteroplasmy number within an individual, adjusting for age and mean nuclear sequencing coverage (*β* = −4.34, *P* = 0.007). Individuals harboring higher numbers of heteroplasmies were more likely to have low mtDNA copy number

We next tested the correlation between mtDNA copy number and heteroplasmy. Since most heteroplasmies were unique to only one individual, especially those with high pathogenic potentials, instead of testing each single mtDNA heteroplasmy, our analysis was restricted to test the association between mtDNA copy number and the total number of heteroplasmies within an individual. With increasing heteroplasmy number, mtDNA copy number significantly decreased (Fig. 4. *β* = −4.34, *P* = 0.007, linear regression, adjusted for age and average nuclear DNA sequencing coverage).

We also tested whether single mtDNA homoplasmic variants were associated with copy number. We identified 186 unique homoplasmic single nucleotide variants, each presented in >1% of individuals in this study population. The associations between mtDNA copy number and these variants were tested using a linear model including age and mean nuclear DNA sequence coverage as covariates. After Bonferroni correction, none of these homoplasmic variants were significantly associated with mtDNA copy number (Additional file 1: Figure S8). Ridge et al. previously reported that 3 mtDNA variants (A9667G, T5277C and C6489A), belonging to haplogroups T2 and U5A1, were significantly associated with higher mtDNA copy number [42]. There were 26 individuals in our dataset harboring A9667G, but this variant was not associated with mtDNA copy number in our test (*P* = 0.5669). The other two variants were missing or found at a rare frequency (7 individuals) in our study, and thus were excluded from further association analysis.

### Phenotypic associations of mtDNA copy number and heteroplasmy load

**Fig. 5:**
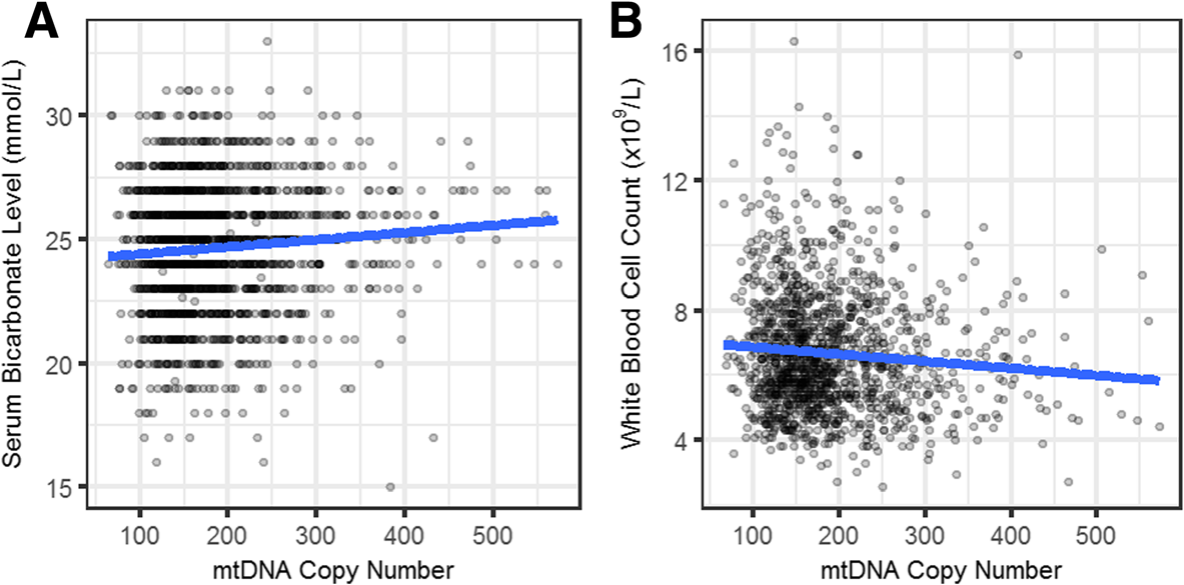
mtDNA copy number association with phenotypic traits. **a** mtDNA copy number was positively associated with serum bicarbonate level (*P* = 4.46e-5). The reference range for bicarbonate level is 22–29 mmol/L (**b**) mtDNA copy number was negatively associated with WBC count (*P* = 0.0006). The reference range for WBC count is 4.0–11.0 (×10^9^/L). The blue lines represent linear regression lines in each case

Age is the most significant risk factor for several diseases. It is possible that the effects of age on mtDNA copy number and heteroplasmy could mediate these effects via their effects on physiological variables known to be perturbed in disease states and with aging. We examined the associations between 32 phenotypic traits provided by TwinsUK cohort and mtDNA copy number / heteroplasmy load (Additional file 3: Table S5). After correcting for multiple testing, mtDNA copy number was significantly associated with serum bicarbonate level (*P* = 4.46e-5, Fig. 5a) and WBC count (*P* = 0.0006, Fig. 5b).

Bicarbonate is an essential component of the pH buffering system and is indirectly related to mitochondrial oxidative reactions. In our analysis, there was a positive correlation between mtDNA copy number and serum bicarbonate level, such that, for each increase of 1 SD in mtDNA copy number (75.8 copies), serum bicarbonate level increased by 0.102 SD (0.27 mmol/L), indicating a potential interplay between the buffering system and mitochondrial activity. Conversely, WBC showed a significant negative correlation with mtDNA copy number. With each increase of 1 SD in mtDNA copy number, WBC count decreased by 0.116 SD (0.2*10^9^ cell/L). WBC count is related to inflammation and immune senescence, so this observation indicated that mtDNA copy number could be associated with immune function.

Since the majority of the heteroplasmies were present in <1% of individuals in the samples, our ability to test their phenotypic associations were limited. Instead of performing analysis on each single heteroplasmy, we aggregated the heteroplasmic mutation information across the entire mitochondrial genome for each individual, and attempted to test the overall cumulative effects of heteroplasmy on different traits. We used the Sequence Kernel Association Test (SKAT) algorithm, which has been shown to have high statistical power under a variety of conditions [38]. Under SKAT default settings, the population frequencies of the variants were used as testing weights, since rare mutations were more susceptible to being deleterious. Here, because most heteroplasmies were only found in one person, we used the predicted pathogenicity of heteroplasmy (CADD scores) as weights. We tried two different genotype matrices, one taking the heteroplasmy MAF into account, the other only considering whether a site was a heteroplasmy or not, regardless of the MAF. In both cases, after multiple test correction, we observed that mtDNA heteroplasmy load was significantly associated with blood apolipoprotein B (ApoB) level (*P* = 1.33e-5 and 3.73e-6, respectively), but not with other phenotypes. Because ApoB is a component of the lipid transport system linked to cardiovascular disease risk [43–46], this suggested a potential link between mtDNA integrity and physiological lipid regulation.

## Discussion

In this study, we first identified mtDNA heteroplasmies in 1511 generally healthy women using PBMC whole genome sequencing data from the UK10K project TwinsUK cohort, with an age range from 17 to 85 years of age. With 2% MAF cutoff, we demonstrated that more than half of the individuals (52.5%) harbor at least one heteroplasmy in their mitochondrial genome, and on average each individual had ~0.9 heteroplasmy. Both the proportion of individuals harboring heteroplasmy and the average heteroplasmy number per person (using the same MAF 2% cutoff) were lower compared to our previous heteroplasmy study using sequencing data from the 1000 genome project, which utilized lymphoblastoid cell lines as source of DNA [8]. This difference could be caused by the difference between the sources of biological material: PMBCs versus cell lines. In cell line transformation, only a small proportion of original cells are induced, hence the heteroplasmy identified in a cell line only represents the heteroplasmy pattern for a few cells instead of the whole cell populations [26]. Because the cell line and PBMC samples show big differences in mtDNA characteristics (Additional file 1: Figure S1), our study reinforces the notion that using DNA directly extracted from human samples is ideal for studying the impact of aging on mtDNA heteroplasmy and copy number. It is also important to point out that our analysis is limited to PBMCs, which are a mixture of different cell types. Further investigation of mtDNA characteristics in these different cell types and their relationship with aging will enable a better understanding on how mtDNA changes during aging.

We observed that heteroplasmy was not distributed uniformly across the mitochondrial genome, and several regions had enriched heteroplasmy: 1. The control region had the highest length normalized occurrence frequency; 2. Mutations located in tRNA-Thr had high occurrence frequency in both heteroplasmy and homoplasmy; 3. Notably, the normalized occurrence frequency of heteroplasmy in the ND5 gene was significantly higher than other protein coding genes, while the frequency of homoplasmy in ND5 was comparable to other genes. mtDNA mutations in the tRNA-Thr and ND5 regions have been reported to be implicated in diseases including Leigh syndrome, mitochondrial myopathy, Parkinson’s disease and thyroid cancer [36]. The high occurrence of mtDNA heteroplasmy in those regions may be a potential source of future diseases, or could reflect an underlying prodromal disease state that independently promotes the accumulation of mtDNA defects.

Using CADD score as a measure of pathogenicity, we observed higher pathogenic potential of mtDNA heteroplasmy compared to homoplasmy, although still lower than disease-associated mutations. We further grouped heteroplasmies by their MAF, and found that heteroplasmies with lower MAF were more pathogenic than the ones with higher MAF, implying that the selective pressure on highly pathogenic heteroplasmies could be stronger, which could occur during germline selection, and hence reduce those heteroplasmies to low frequency. Due to mitochondrial threshold effects [47], highly-pathogenic heteroplasmies can persist in healthy individuals at low frequency; however, once they reach high frequency, they could potentially contribute to mitochondrial dysfunction and further lead to the onset and/or progression of various age-related diseases, as previously suggested [48–50].

It has been proposed that patients with mitochondrial diseases experience a monoclonal expansion of a single deleterious mtDNA mutation (for example, 3243A > G), whereas aging is associated with a mosaic of multiple low-level mtDNA mutations accumulated during a lifetime [51]. mtDNA is replicated throughout the lifetime of an individual, independent of cell cycle. Both inherited and de novo mutations that emerged early in life could clonally expand to increase the heteroplasmy burden over time in a sub-population of cells. Several studies have reported that the amount of mtDNA mutation increases with age in several human tissues, including muscle, colon, putamen and heart [52–55]. Consistent with these reports, we observed that the heteroplasmy burden was elevated in older individuals. In the current sample, the absolute heteroplasmy number increased by 58.5% in individuals over 70-years (mean age 74.04) compared to individuals under 40-years (mean age 30.12). Meanwhile there was a trend for an increasing proportion of nonsynonymous heteroplasmy in older individuals. Given that the individuals involved in this study were generally healthy, it is possible that in aged individuals with diseases, more pronounced increases of heteroplasmy burden and pathogenicity would be observed. Future large-scale prospective studies should investigate the relationship between mtDNA heteroplasmy, disease status, and mortality.

Besides mtDNA quality, mtDNA quantity has also been suspected to be influenced by age. We estimated mtDNA copy number using WGS data, and found that mtDNA copy number was negatively correlated with age. These age-related mtDNA copy number changes were also reported in other studies. Wachsmuth et al. suggested that mtDNA copy number decreased with age in human muscle tissue [37] and Sahin et al. found a similar decrease in mice and rats in myocardial, hepatic, and hematopoietic cells [56, 57]. In measurements from whole blood, which are potentially confounded by several factors, Mengel-From et al. also reported a decline of 5.4 copies per decade of life in individuals above 48 years old [12].

However, no studies have investigated whether the effects of age on the two mitochondrial characteristics are independent, as it is possible that age can affect mtDNA copy number through age-related heteroplasmy changes or vice versa. In this study, we demonstrated that age was independently associated with mtDNA copy number and heteroplasmy. Furthermore, compared to previous studies, we also included WBC count and platelet count as covariates in the regression model to adjust for potential bias caused by blood cell contaminations. Mitochondrial biogenesis has been proposed as a marker of many age-related health outcomes or even the aging process itself [58]. Our results suggested that both mtDNA heteroplasmy and copy number should be included to establish this relationship. Mitochondrial mutations that occur early in life can clonally expand to cause mitochondrial dysfunction and further contribute to aging through a number of potential mechanisms including decreased oxidative capacity and energy production capacity, but also nuclear signaling and transcriptional dysregulation [59–63]. In addition, decreased mtDNA copy number may also lead to decreased energy production and/or decreased mitochondrial gene expression [57, 64]. Maintaining both mtDNA quality and quantity together may help to counteract or slow down the aging process.

Our data were also consistent with the idea that mtDNA copy number and heteroplasmy can influence each other. We observed a negative correlation between mtDNA copy number and total number of heteroplasmies in an individual. In mitochondrial diseases, a compensatory increase in mtDNA copy number via mitochondrial biogenesis may effectively compensate for heteroplasmic mtDNA mutations and mitochondrial dysfunction [65–67]. Thus, the observed age-related copy number decrease may result in a weaker copy number buffering effect during aging. In contrast, our results suggest that mtDNA haplogroups and homoplasmic variants were not strongly associated with mtDNA copy number. Although haplogroup T2 has been reported to be associated with higher mtDNA copy number [42], we did not observe this association in our dataset. This may be caused by different sample sizes for this specific haplogroup. Our data had 177 individuals belonging to T2 while the conclusions in the previous study [42] only included 12 individuals. Another study suggested that haplogroup J had higher copy number compared to haplogroup H [68]. However, neither Wachsmuth et al. [37] nor our data found this difference. It should be noted that our dataset only included UK females of European descent. To identify potential haplogroup-related effects on mtDNA copy number, further studies are needed to include a more ethnically-diverse range of populations, with both men and women, and larger sample sizes.

These age-related mitochondrial changes, combined with the fact that age is the main risk factor of many diseases in the population [69], further directed us to investigate mitochondrial associations with human physiological traits. After controlling for age, we found that serum bicarbonate level and WBC count were significantly associated with mtDNA copy number. The bicarbonate-carbon dioxide buffer system in blood can influence the pH gradient across the inner membrane of mitochondria, and thus may provide a link between systemic acid-base balance and regulation of mitochondrial metabolism [70, 71]. It has also been reported that reducing muscle hydrogen ion accumulation by sodium bicarbonate during running training was associated with greater improvements in both mitochondrial mass and mitochondrial respiration in rat models [72]. Our result was consistent with these reports and suggests a potential interplay between the bicarbonate buffer system and mitochondrial biogenesis with aging.

WBC count is a well-established marker for inflammation [73, 74]. Its negative correlation with mtDNA copy number indicated a potential change in mitochondrial biogenesis during the immune response. Decreased peripheral blood mtDNA copy number is observed in various diseases accompanied with inflammation, for example, COPD was associated with decreased leukocyte mtDNA copy number [75]. Decreased mtDNA copy number was also observed to be significantly associated with adverse clinical outcomes in peritoneal dialysis patients [76]. Mitochondria play an important role in inflammatory signaling; conversely, inflammation may also damage mtDNA, promoting a vicious inflammatory cycle [77]. However, because WBCs are a mixture of different immune cells, a change in the composition of different immune cells, or all immune cell types undergo similar age-related changes in mtDNA copy number, may both contribute to the decrease of mtDNA copy number detected here. Further studies are needed to elucidate this observation. The link to specific pro- and anti-inflammatory biomarkers will also be important to resolve.

Most heteroplasmic variants had very low frequency in the population, which limited our ability to test for associations. Inspired by studies on nuclear DNA rare variants [78, 79], instead of evaluating single variants, we aggregated heteroplasmic mutations across the entire mitochondrial genome as a “heteroplasmy mutation load”, and tested the association between this mutation load and different healthy traits. By applying the SKAT algorithm, we found that mtDNA heteroplasmy load was significantly associated with blood ApoB level independent of age. Mitochondria play a critical role in fatty acid metabolism (eg, β-oxidation). Furthermore, ApoB is the main structural surface protein found on all beta-lipoproteins, which is important for lipid transportation. The ApoB level is predictive for atherosclerosis [80], and the onset of obesity is usually accompanied by overproduction of ApoB [81]. Our result suggests a potential interaction between mitochondrial function and ApoB metabolism. It has been reported that the suppression of the PPARα signaling pathway would result in disrupted mitochondrial integrity and upregulated hepatic *apoB* gene expression at both the transcriptional and translational level in liver [82], providing a potential mechanism for how mitochondrial dysfunction is connected with ApoB metabolism. Nonetheless, further studies are needed to elucidate this connection.

One limitation of our study is that all participates were female. Given sex differences in mtDNA copy number measured in whole blood [24, 25, 83], our findings may not be representative for both men and women. In a study of whole blood, mtDNA copy number was previously reported to be associated with waist circumference and waist-hip ratio, suggesting an association between mtDNA copy number and fat distribution and lipid metabolism [25]. In our study, we did not observe these associations, which could possibly be caused by sex differences, or by other confounding factors (platelets, cell-free DNA, or other) in previous studies compared to purified leukocytes in this study.

## Conclusion

In conclusion, using WGS data from the UK10K project TwinsUK cohort, we conducted, to date, the first study addressing whether the age effects on mtDNA heteroplasmy and copy number are independent. Our analyses reveal that mtDNA copy number is inversely correlated with heteroplasmy number, and associated with serum bicarbonate level and WBC count. Moreover, heteroplasmy load is associated with blood ApoB level, suggesting future avenues for research aimed at understanding the role of mitochondrial dysfunction in human aging. Mitochondria play a central role in cellular energy metabolism and regulate a broad range of cellular activities, and alterations of mtDNA sequence integrity and copy number have been implicated in human disease. Therefore, it remains promising to further investigate whether approaches to maintain mtDNA copy number and manage the expansion of mtDNA heteroplasmic mutations could help improve health status, especially in the elderly.

## Additional files

Additional file 1: Supplementary Tables S2 to S4, Figures S1 to S8. (DOCX 4879 kb)

Additional file 2: Table S1. Heteroplasmy information. (CSV 49 kb)

Additional file 3: Table S5. Associations between mtDNA copy number and 32 phenotypic traits. (XLSX 11 kb)

## Abbreviations

ALSPAC: Avon longitudinal study of parents and children
ApoB: Apolipoprotein B
CADD Score: combined annotation-dependent depletion score
MAF: Minor allele frequency
mtDNA: Mitochondrial DNA
nDNA: Nuclear DNA
NUMT: Nuclear mitochondrial DNA
PBMC: Peripheral blood mononuclear cell
SKAT: Sequence kernel association test
WGS: Whole genome sequencing
