# Supplementary material for: Independent impacts of aging on mitochondrial DNA quantity and quality in humans
Source: BMC Genomics. 2017 Nov 21;18:890. doi: 10.1186/s12864-017-4287-0 (PMC5697406; doi:10.1186/s12864-017-4287-0)
Supplement: Supplementary file 1 — Supplementary Tables S2 to S4, Figures S1 to S8. (DOCX 4879 kb) [file 12864_2017_4287_MOESM1_ESM.docx]

**Independent Impacts of Aging on Mitochondrial DNA Quantity and Quality in Humans**

Ruoyu Zhang^a^, Yiqin Wang^a^, Kaixiong Ye^b^, Martin Picard^c^, Zhenglong Gu^a^

^a^ Division of Nutritional Sciences, Cornell University, Ithaca, New York 14853, USA

^b^ Department of Biological Statistics and Computational Biology, Cornell University, Ithaca, New York 14853, USA

^c^ Department of Psychiatry, Division of Behavioral Medicine, Department of Neurology and Columbia Translational Neuroscience Initiative, Columbia Aging Center, Columbia University Medical Center, New York, NY 10032 USA

**Emails:**

Ruoyu Zhang: [rz253@cornell.edu](mailto:rz253@cornell.edu)

Yiqin Wang: [yw729@cornell.edu](mailto:yw729@cornell.edu)

Kaixiong Ye: [ky279@cornell.edu](mailto:ky279@cornell.edu)

Martin Picard: [mp3484@columbia.edu](mailto:mp3484@columbia.edu)

Zhenglong Gu: [zg27@cornell.edu](mailto:zg27@cornell.edu)

**Corresponding Author:** Zhenglong Gu

312 Savage Hall, Cornell University, Ithaca, NY 14853

Phone: 607-254-5144 Fax: 607-255-1033

Email: [zg27@cornell.edu](mailto:zg27@cornell.edu)

**Supplementary information**

**Table S1. Heteroplasmy information.**

See Table S1.csv file

**Table S2. Normalized occurrence frequency of heteroplasmy and homoplasmy in different functional groups and two genes with high occurrence frequencies.**

|  | Average Normalized Occurrence Frequency | |
| --- | --- | --- |
|  | Heteroplasmy | Homoplasmy |
| Intergenic | 0 | 0 |
| Control Region | 0.000206 | 0.000355 |
| rRNA | 0.000044 | 0.000080 |
| tRNA | 0.000026 | 0.000022 |
| Protein Coding | 0.000049 | 0.000032 |
|  | | |
| tRNA-Thr | 0.000124 | 0.000163 |
| ND5 | 0.000096 | 0.000038 |

**Table S3. Correlation of age with mtDNA heteroplasmy number and copy number (Down-sampled data).**

| Parameter | Parameter Estimate | SE | P Value |
| --- | --- | --- | --- |
| mtDNA heteroplasmy number | 1.067 | 0.278 | 0.000129 *** |
| mtDNA copy number | -0.012 | 0.004 | 0.004833 ** |

Significance level (* *P* < 0.05, ** *P* < 0.01 and *** *P* < 0.001)

**Table S4. Correlation of age with mtDNA heteroplasmy number and copy number, adjusting for WBC and platelet counts (Down-sampled data).**

| Parameter | Parameter Estimate | SE | P Value |
| --- | --- | --- | --- |
| mtDNA heteroplasmy number | 0.789 | 0.258 | 0.002253 ** |
| mtDNA copy number | -0.015 | 0.004 | 0.000233 *** |
| White blood cell count | -0.319 | 0.176 | 0.070713 |
| Platelet count | 0.009 | 0.005 | 0.088370 |

Significance level (* *P* < 0.05, ** *P* < 0.01 and *** *P* < 0.001)

**Table S5. Associations between mtDNA copy number and 32 phenotypic traits.**

See Table S5.xlsx file

**Fig S1.**


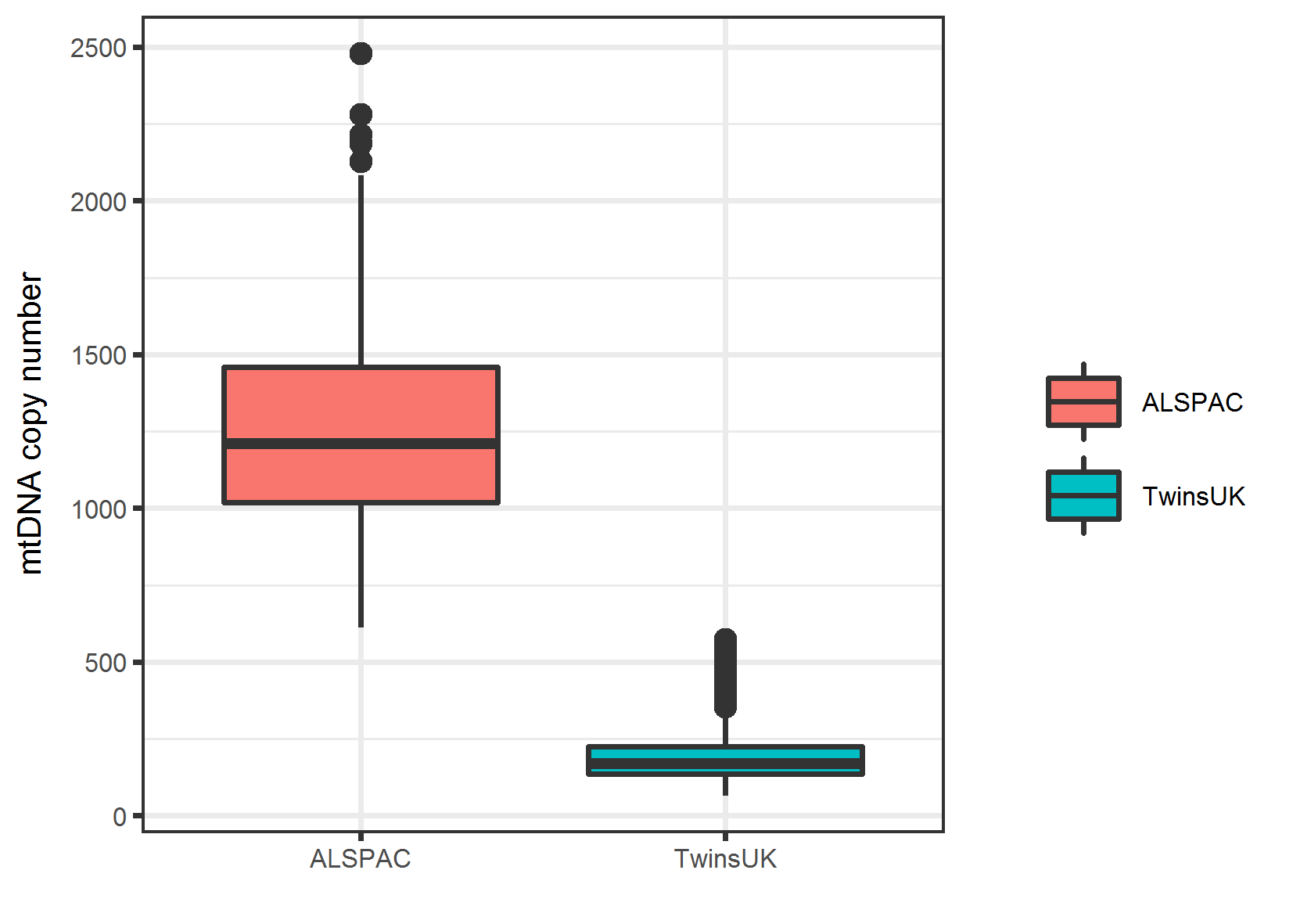


**mtDNA copy number comparison between cell lines and PBMCs DNA.** mtDNA copy number was estimated in two UK10K cohorts, ALSPAC (DNA extracted from cell line) and TwinsUK (DNA extracted from PBMCs). On average, mtDNA copy number in cell lines was 5~10-fold higher than in PBMCs.

**Fig S2.**

**
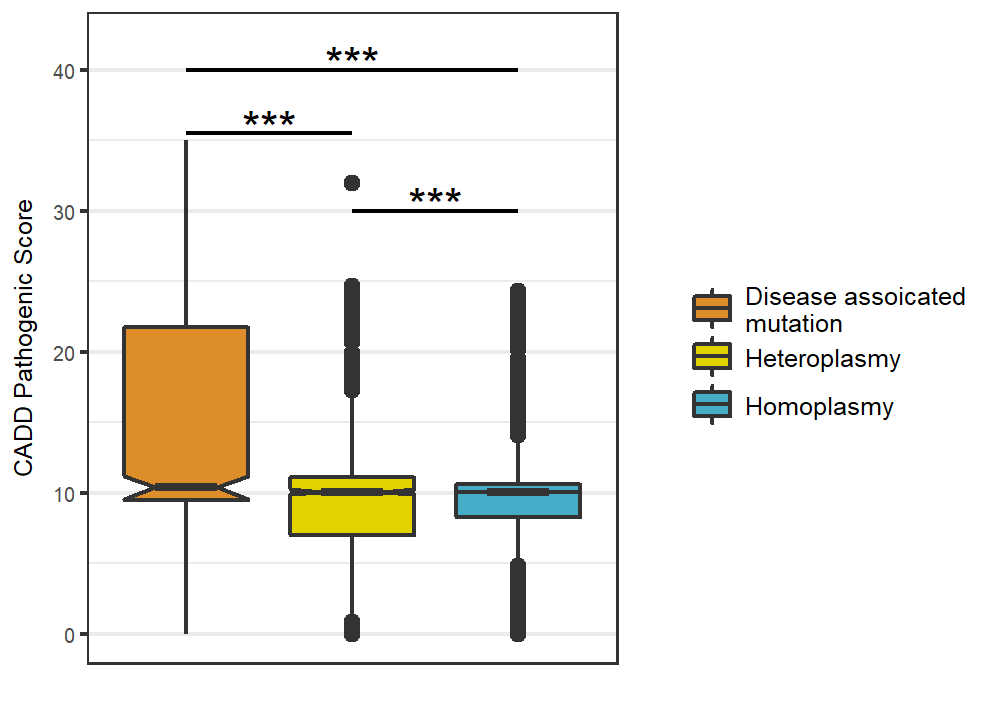
**

**Pathogenic potential for all heteroplasmies.** The box plot of CADD pathogenic score for disease associated mutations, heteroplasmies and homoplasmies. Heteroplasmies had significantly higher pathogenic scores than homoplasmies (*P* = 3.566e-11).

**Fig S3.**


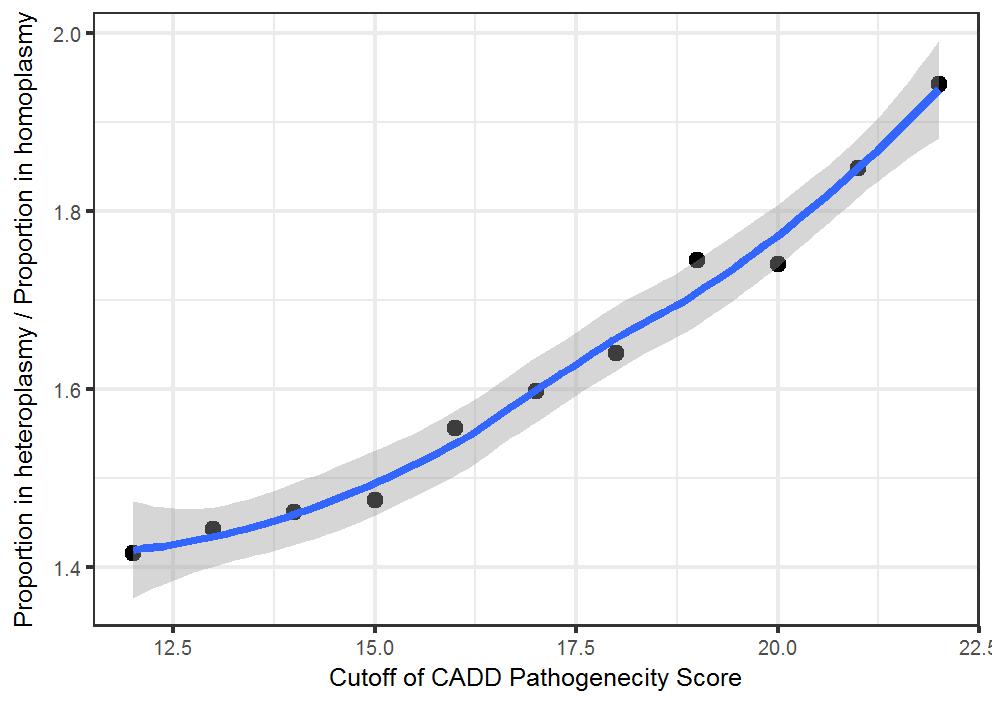


**Ratio of heteroplasmic to homoplasmic pathogenic mutations under different CADD score cutoffs.** A pathogenic mutation was recognized if its CADD score exceeded a certain cutoff. The proportion of pathogenic mutation in heteroplasmy was larger than that in homoplasmy under different cutoffs. Heteroplasmy was 1.42 to 1.94 times more likely to be pathogenic than homoplasmy under different cut offs.

**Fig S4.**

**
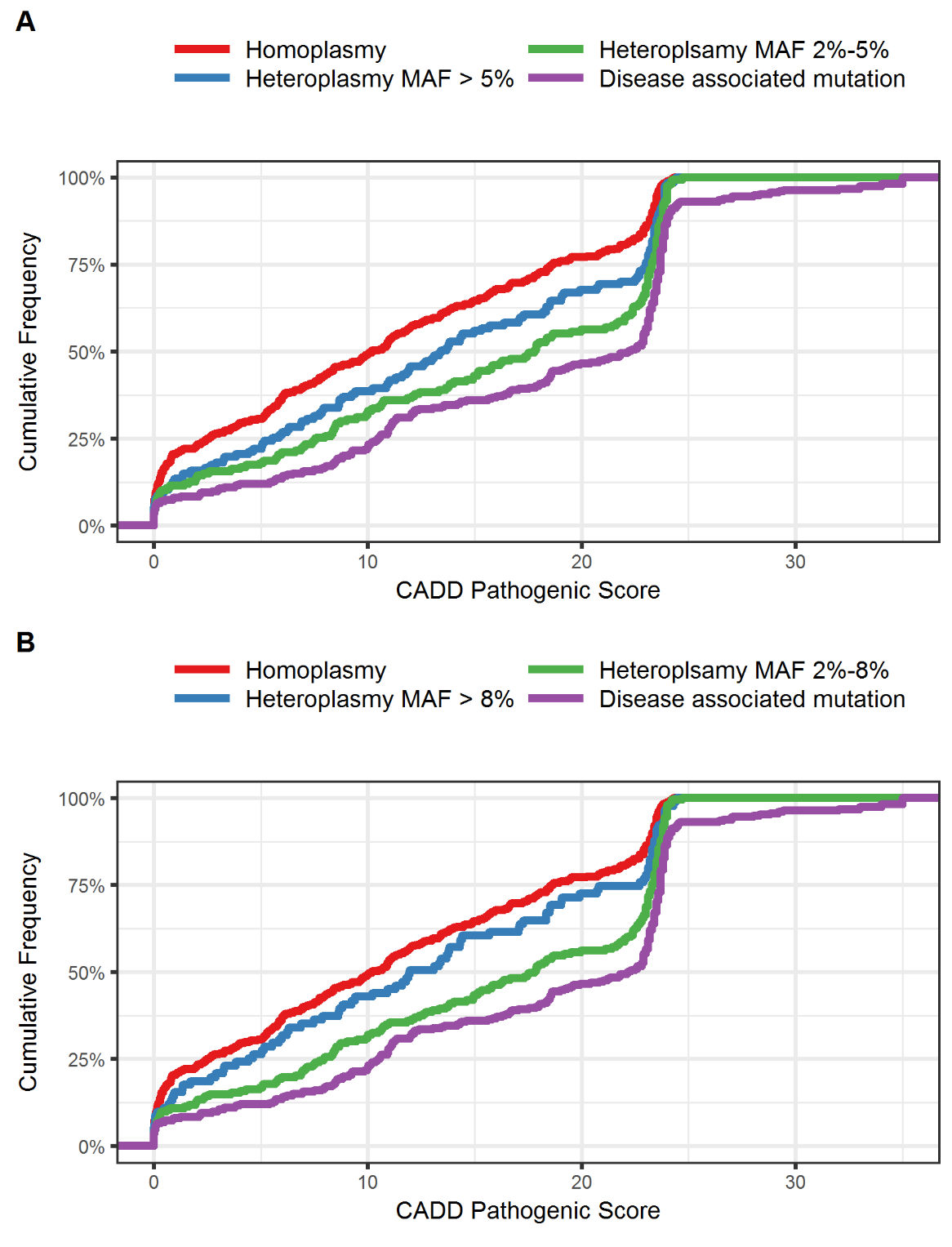
**

**Pathogenic potentials for nonsynonymous heteroplasmy.** The cumulative distribution of pathogenic scores for disease associated mtDNA mutations, homoplasmy, low frequency heteroplasmy and high frequency heteroplasmy. The low frequency and high frequency heteroplasmy were separated by two different arbitrary cutoffs (others are the same): (A) low frequency 2-5%, high frequency >5%. (B) low frequency 2%-8%, high frequency >8%. In both (A) and (B), the distribution of low frequency heteroplasmy was closer to disease associated mutations, indicating higher pathogenic potentials.

**Fig S5.**


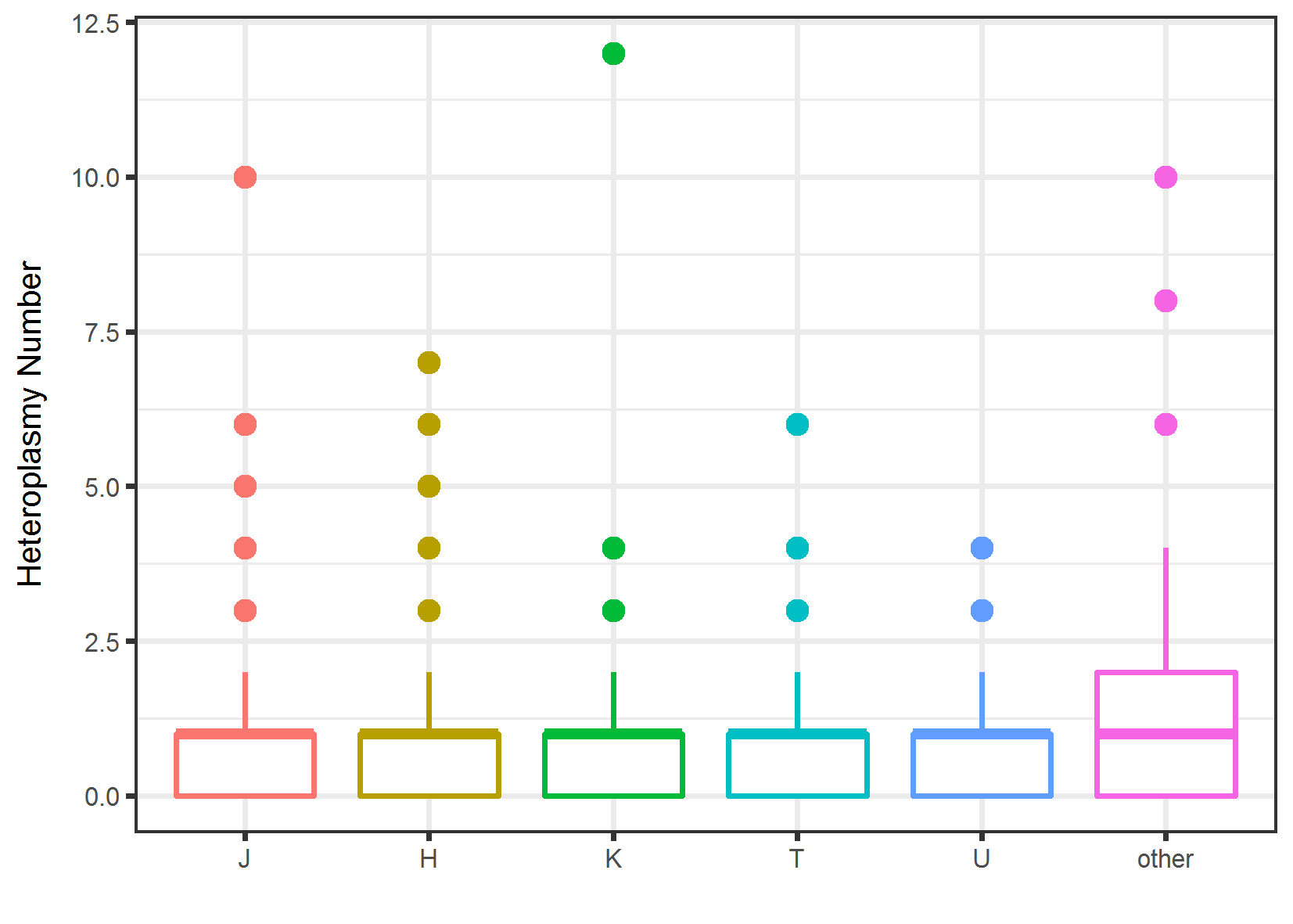


**Boxplot of mtDNA heteroplasmy number within an individual in different haplogroups.** mtDNA heteroplasmy number was not significantly affected by haplogroup.

**Fig S6**

**
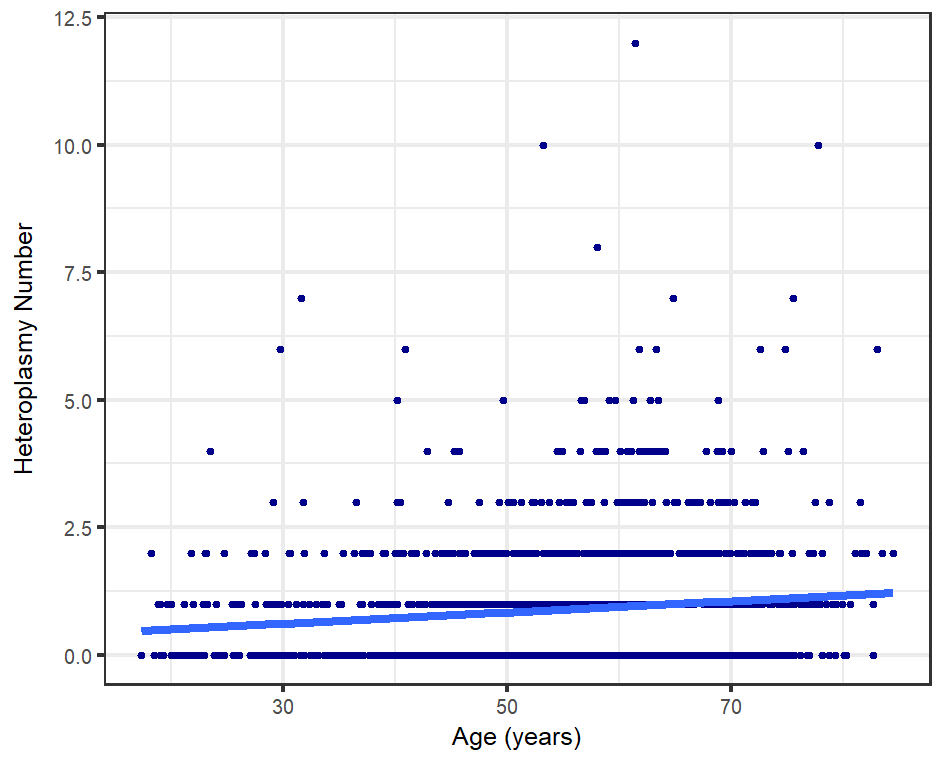
**

**Association between mtDNA heteroplasmy number and age.** mtDNA heteroplasmy number was significantly associated with age (*β* = 0.011, *P* = 5.77e-6,). The blue line represents a linear regression line.

**Fig S7.**


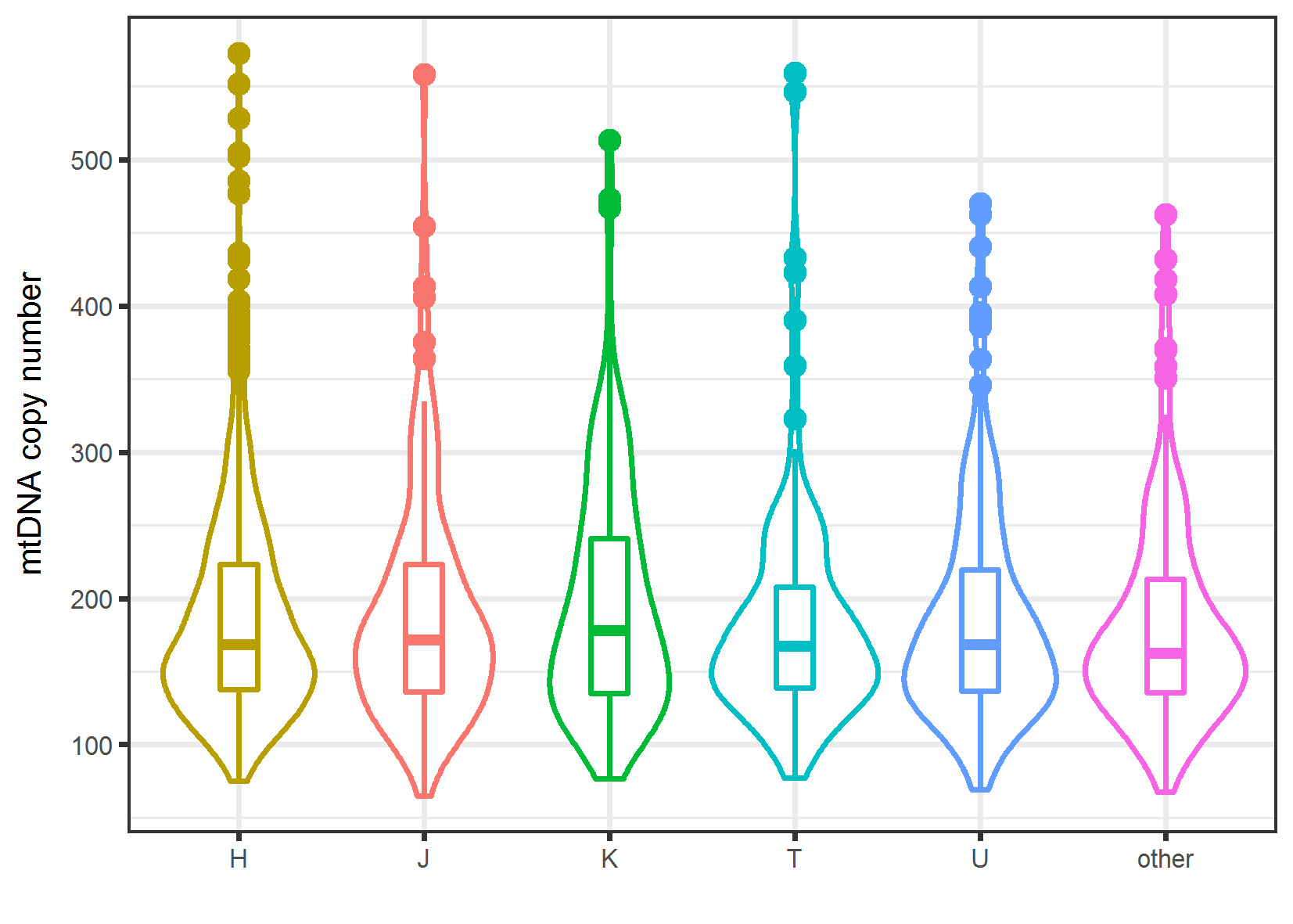


**Boxplot of mtDNA copy number in different haplogroups.** mtDNA copy number was not significantly affected by haplogroup.

**Fig S8.**


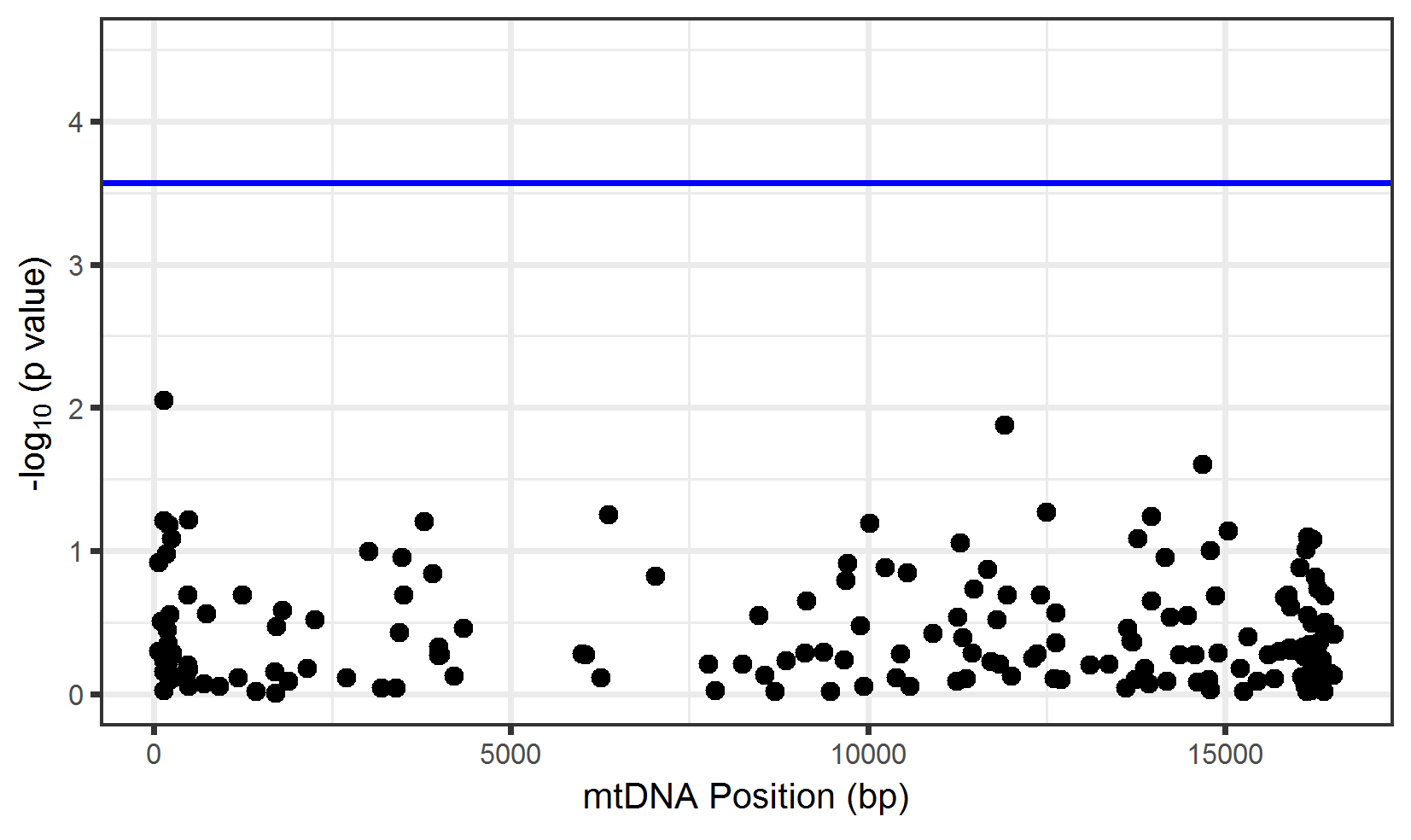


**Manhattan plot of associations between homoplasmic variants and mtDNA copy number.** The Blue horizontal line indicates the mtDNA genome wide significance threshold (*P* = 2.69e-4). No significantly associated homoplasmy was detected in this analysis.
